# Editorial: Physical exercise and brain health: functional mediators and therapeutic targets focusing on neuroendocrinology

**DOI:** 10.3389/fnins.2024.1415752

**Published:** 2024-04-25

**Authors:** Weina Liu, Laura Piccardi, Howe Liu, Li Zhang, Chengyi Liu, Jie Xia

**Affiliations:** ^1^Key Laboratory of Adolescent Health Assessment and Exercise Intervention of Ministry of Education, East China Normal University, Shanghai, China; ^2^College of Physical Education and Health, East China Normal University, Shanghai, China; ^3^Department of Psychology, Faculty of Medicine and Psychology, Sapienza University of Rome, Rome, Italy; ^4^San Raffaele Cassino Hospital, Cassino, Italy; ^5^Department of Physical Therapy, School of Allied Health Professions, Louisiana State University Health Science Center, New Orleans, LA, United States; ^6^Guangdong-Hongkong-Macau Institute for CNS Regeneration, Jinan University, Guangzhou, China; ^7^School of Physical Education and Sports Science, South China Normal University, Guangzhou, China; ^8^Department of Physical Education, Shanghai Jiao Tong University, Shanghai, China

**Keywords:** physical exercise, brain health, neuroendocrine, exerkines, neuropsychiatric disorders

## Introduction

In the effort to address neuropsychiatric disorders, particularly in the context of the increasing prevalence of metabolic diseases and brain-related conditions, the significance of physical exercise as a valuable adjunct becomes apparent. With its diverse and comprehensive impact on the neuroendocrine system, physical exercise presents a promising opportunity for intervention and treatment. By exploring the intricate dynamics of this relationship, we can gain a deeper understanding of how exercise revolutionizes our strategies for mental health care. In this Research Topic of works, we present six studies focusing on enhancing comprehension of the intersection between neuroendocrinology and physical exercise within the realm of brain diseases.

## Central targets of metabolic diseases

Cognitive dysfunction often occurs with metabolic diseases like diabetes and obesity. Studies suggest that exercise can help reduce the risk of cognitive impairment in these conditions, but the reasons are not fully understood. The studies conducted by Moreno et al. and He et al. provide valuable insights into the significant influence of physical activity on metabolic disorders and cognitive health. Moreno et al. demonstrate a positive association between heightened physical activity levels and greater gray matter volumes in individuals with type 2 diabetes, indicating a potential safeguarding effect on brain morphology. Likewise, He et al. emphasize the importance of exercise in ameliorating cognitive impairments linked to obesity, partially mediated by insulin-like growth factor 1 (IGF-1). These insights highlight the neuroprotective benefits of exercise in combating the cognitive repercussions of metabolic diseases.

## Central targets of brain diseases

It is widely recognized that exercise significantly impacts on neural signaling molecules within the brain. Nevertheless, the precise alterations in neural signaling molecules induced by exercise in various diseases are still not discerned, highlighting the need for a more nuanced differentiation. Hu et al.'s review underscores the necessity for further investigation into the influence of exercise on adolescent mental health, aligning with the demand for methodologically robust studies to elucidate the underlying neurobiological mechanisms. Likewise, Luthra et al.'s research illuminates the diverse impacts of exercise on hormone regulation in Parkinson's disease, suggesting its potential to alleviate both motor and non-motor symptoms by modulating cortisol, melatonin, insulin, and IGF-1 levels. These insights emphasize exercise's role as a modulator of central neurochemistry, offering avenues for targeted interventions in various brain disorders.

## Peripheral targets of brain diseases

Exercise enhances brain function through the regulation of molecules such as metabolites, cytokines, and hormones originating from peripheral tissues and organs. This novel perspective in integrative physiology elucidates the mechanisms by which exercise protects the brain. Skeletal muscle is increasingly recognized as a significant endocrine organ that secretes substances like irisin and cathepsin B during exercise, offering neuroprotective advantages. Nevertheless, our understanding of these muscle-derived factors remains incomplete. Ataka et al.'s study on musclin and its impact on depression highlights the promise of targeting peripheral mechanisms in mental health treatment. Their findings demonstrate that musclin administration can mitigate depressive symptoms in mice by modulating the levels of Ucn 2 and proopiomelanocortin in the hypothalamus. Additionally, the researchers observed changes in c-Fos-positive cell activity in specific brain regions, such as the paraventricular nucleus and nucleus tractus solitarii, suggesting a potential role for musclin in regulating neural activity related to depression. This study illuminates a muscle-brain crosstalk mechanism, highlighting the crucial role of muscles as endocrine organs in mediating the promotion of brain health through exercise.

## Psychosocial effect of exercise

The biological mechanisms of exercise receive significant attention, yet it is also imperative to consider the social and psychological benefits of exercise. Factors such as mood and sleep, which are intricately linked to cognitive function and mental health, can be positively impacted by exercise interventions. Zhai et al.'s study on female sex workers underscores the broader psychosocial implications of exercise on mental health. While highlighting risk factors such as isolation and pandemic-related stressors, it stresses the protective effect of social support networks and regular physical activity. By recognizing the interconnectedness of biological, psychological, and social factors, interventions can be tailored to address the holistic needs of vulnerable populations, fostering resilience and wellbeing.

## Summary and future perspectives

In summary, this Research Topic of works has provided valuable insights into the mechanisms underlying the effects of exercise on combating neuropsychiatric disorders from a neuroendocrine perspective. These findings underscore the neuroendocrine system as a rich source of exerkines, offering promising avenues for investigating exercise interventions in neurological disorders and the potential advancement of exercise-based therapies. In fact, the current opinions of physical exercise in brain health mainly argue for the role of selected exerkines in modulating brain functions. Although numerous exerkines have been identified, our understanding of the peripheral-central network under the exercise paradigm is far from complete, as several critical questions have not been resolved. The first and foremost question is the effectiveness of each of exerkines under physiological conditions. Current research predominantly concentrates on isolated molecules in rodent models, indicating a need to transition toward more comprehensive methodologies to clarify the crosstalk among multiple exerkines. The second and third question calls for the origin and modulatory mechanism of peripheral exerkines. Although rodent models are frequently utilized due to ethical considerations, non-invasive imaging methodologies for human investigations are imperative for monitoring exerkine dissemination. Furthermore, it is imperative to engage in interdisciplinary collaborations among the fields of neuroscience, physiology, molecular biology, and sports science to thoroughly investigate the interaction between the brain and other organs during different exercise regimens. This approach will enhance our comprehension of the effects of exercise on brain function and its potential therapeutic applications for neurological disorders.

## Author contributions

WL: Conceptualization, Writing – original draft, Writing – review & editing. LP: Writing – original draft, Writing – review & editing. HL: Writing – original draft, Writing – review & editing. LZ: Writing – original draft, Writing – review & editing. CL: Writing – review & editing. JX: Writing – original draft, Writing – review & editing.

